# Multi-Omic Atlas reveals cytotoxic phenotype and ROS-linked metabolic quiescence as key features of CTL-resistant HIV-infected CD4 ^+^ T-cells

**DOI:** 10.1101/2024.12.22.629960

**Published:** 2024-12-23

**Authors:** Alberto Herrera, Louise Leyre, Jared Weiler, Noemi Luise Linden, Tan Thinh Huynh, Feng Wang, Colin Kovacs, Marina Caskey, Paul Zumbo, Maider Astorkia Amiama, Sandra Terry, Ya-Chi Ho, Doron Betel, R. Brad Jones

## Abstract

Cytotoxic T-lymphocytes (CTL) exert sustained pressure on reservoirs of HIV-infected cells that persist through years of antiretroviral therapy (ART). This selects for latently infected cells, but also potentially for cells that express HIV but possess intrinsic CTL resistance. We demonstrate that such resistance exists in HIV-infected CD4 ^+^ T-cells that survive rigorous CTL attack and map CTL susceptibility to cell identities and states defined by single-cell multi-omics and functional metabolic profiling. Cytotoxic CD4 ^+^ T-cells were prominently overrepresented amongst survivors, as were cells with quiescent metabolic profiles and low levels of reactive oxygen species (ROS) production. The induction of ROS production by treatment with deferoxamine sensitized these cells to CTL-mediated elimination. Reservoir-harboring cells from clinical samples share the above transcriptional features, being enriched for quiescent states. Our results provide an atlas for elucidating features of CTL resistance in HIV reservoirs, and identify oxidative stress as a therapeutic target to facilitate reservoir elimination.

## Full Text Availability

The license terms selected by the author(s) for this preprint version do not permit archiving in PMC. The full text is available from the preprint server.

